# Delayed small bowel octreotide response in patients with hereditary transthyretin amyloidosis

**DOI:** 10.1186/1750-1172-10-S1-P36

**Published:** 2015-11-02

**Authors:** Wixner Jonas, Ole Suhr, Pontus Karling, Greger Lindberg

**Affiliations:** 1 Umeå University, Dept of Public Health and Clinical Medicine, 90187, Umeå, Sweden; 2 Karolinska University Hospital Huddinge, Dept of Medicine, 14186, Stockholm, Sweden

## Background

Gastrointestinal (GI) complications such as constipation, diarrhea and gastroparesis are common in hereditary transthyretin (ATTR) amyloidosis. The mechanisms behind these disturbances have not been fully elucidated and the patients' small bowel function remains largely unexplored. The aim of the present study was to compare the small bowel motility in patients with hereditary ATTR amyloidosis with that in non-amyloidosis controls.

## Methods

Ambulatory 24-hour small bowel manometries were performed at Karolinska University Hospital, Huddinge, Sweden. Jejunal recording sites and standardized meals were used during the tests. The somatostatin analogue octreotide (50 µg subcutaneously) was used for inducing fasting motility two hours after the last test meal (breakfast). Patients with hereditary ATTR amyloidosis undergoing evaluation for liver transplantation were consecutively selected for manometry (n = 19), and for each patient three age and gender matched controls (n = 57) with functional GI disorders were selected for comparison. Patients with an age at onset of 50 years or more were defined as late-onset cases. Non-parametrical tests were used for all statistical analyses.

## Results

The patients' median age at examination was 52.8 (30.8-66.5) years and the median duration of symptomatic disease was 2.3 (0.5-9.7) years. A majority (89%) of the patients carried the V30M mutation, 58% had GI symptoms and 84% had a PND score of I. Small bowel manometry was judged to be normal in 42% of the patients and 74% of the controls (p = 0.01). Patients displayed significantly more phase III migrating motor complexes during day-time than the controls (in median 4 vs. 2, p <0.01), and had a delayed response to octreotide (in median 5.0 min vs. 3.8 min, p = 0.02). Low-amplitude complexes were more common in patients than in controls (16% vs. 4%), however, this difference did not reach statistical significance (p = 0.10). Among the patients, late-onset cases showed a longer delay in octreotide response (in median 5.4 vs. 4.3 min, p = 0.03), but no major difference related to gender, presence of GI symptoms, PND score or TTR mutation was found for any of the variables.

## Conclusions

Patients with early-stage hereditary ATTR amyloidosis only showed minor abnormalities in their small bowel motility. The main finding was a delayed response to octreotide injection, which may reflect an autonomic neuropathy and changes in the neuroendocrine system of the gut, including a depletion of interstitial cells of Cajal and a reduction of endocrine cells. Surprisingly, late-onset cases had a longer delay in octreotide response, however, this might be an age-related finding.

